# Draft Genome Sequences of the Three *Massilia* Strains AB1, ST3, and ZL223

**DOI:** 10.1128/MRA.00451-21

**Published:** 2021-07-29

**Authors:** Ariel A. Bradley, Zoephia Laughlin, Saralexis Torres, Loralyn M. Cozy

**Affiliations:** a School of Nursing, Illinois Wesleyan University, Bloomington, Illinois, USA; b Department of Biology, Illinois Wesleyan University, Bloomington, Illinois, USA; University of Maryland School of Medicine

## Abstract

To increase the genomic data available for antibiotic discovery, three independently isolated antibiotic-producing *Massilia* strains were sequenced. No more than 84% average nucleotide identity was shared with publicly available *Massilia* genomes, and a low similarity of predicted biosynthetic gene clusters to known clusters was found.

## ANNOUNCEMENT

The bacterial genus *Massilia* (1–3) is found in a wide range of locations (4–7) and has been investigated for the production of interesting compounds (5, 6, 8–16) and metabolic functions (17–23). Here, we sequenced the genomes of three antibiotic-producing *Massilia* isolates, AB1, ST3, and ZL223 (Table 1).

**TABLE 1:** Accession numbers, sequencing features, and genomic characteristics of the *Massilia* strains

Strain	GenBank accession no.	GenBank assembly accession no.	Sequence Read Archive accession no.	Location^*a*^	No. of reads	Read length (bp)	No. of contigs	*N*_50_ (bp)	Avg coverage (×)	Size (bp)	% GC	No. of genes	No. of proteins
AB1	JAGKSG000000000.1	GCA_018119385.1	SRR14249197	40.4926 N, 88.9920 W	2,502,728	35,149	110	107,092	138	5,258,207	66.07	4,804	4,682
ST3	JAGPWC000000000.1	GCA_018119405.1	SRR14249196	40.4928 N, 88.9913 W	2,651,558	35,149	155	67,988	145	5,275,928	66.67	4,765	4,664
ZL223	JAGPWD000000000.1	GCA_018119395.1	SRR14249195	40.4934 N, 88.9929 W	3,502,363	35,149	86	98,998	189	5,362,323	66.10	4,890	4,789

a GPS coordinates of soil collection site (latitude and longitude).

Soil from three independent sites was dilution plated onto Reasoner’s 2A (R2A) solid medium (24), and individual colonies were screened for antibiotic activity. The strains described here were selected because they exhibited a zone of inhibition against the lawn of at least one bacterium tested. Strain AB1 produced a zone against Staphylococcus cohnii. ST3 produced a zone against *S. cohnii*, Bacillus subtilis, Escherichia coli, Pseudomonas putida, Enterobacter aerogenes, and Acinetobacter baylyi. ZL223 produced a zone against *S. cohnii* and A. baylyi. The isolates were identified as *Massilia* by 16S PCR and grown overnight in R2A liquid medium with shaking at 28°C to an optical density at 600 nm (OD_600_) of ∼1.0. Genomic DNA was purified using the Qiagen DNeasy blood and tissue kit and submitted to the Microbial Genome Sequencing Center. Libraries were prepared with a small-volume tagmentation protocol using the Illumina Nextera DNA library kit. Using PCR with the KAPA HiFi library amplification kit, the remaining adapters and barcodes were attached and the library was amplified (25). The library was sequenced on an Illumina NextSeq 550 instrument. The quality of the resulting adapter-trimmed paired-end reads was assessed using FastQC v0.11.9 (26) with default parameters. The reads were assembled within PATRIC v3.6.9 (27) using Unicycler v0.4.8 (28), polished using two Pilon iterations (29), and analyzed using QUAST (30), all with default parameters. Annotation was performed using the Prokaryotic Genome Annotation Pipeline v5.1 using the MIGS soil package (31, 32) and NCBI parameters.

**FIG 1:**
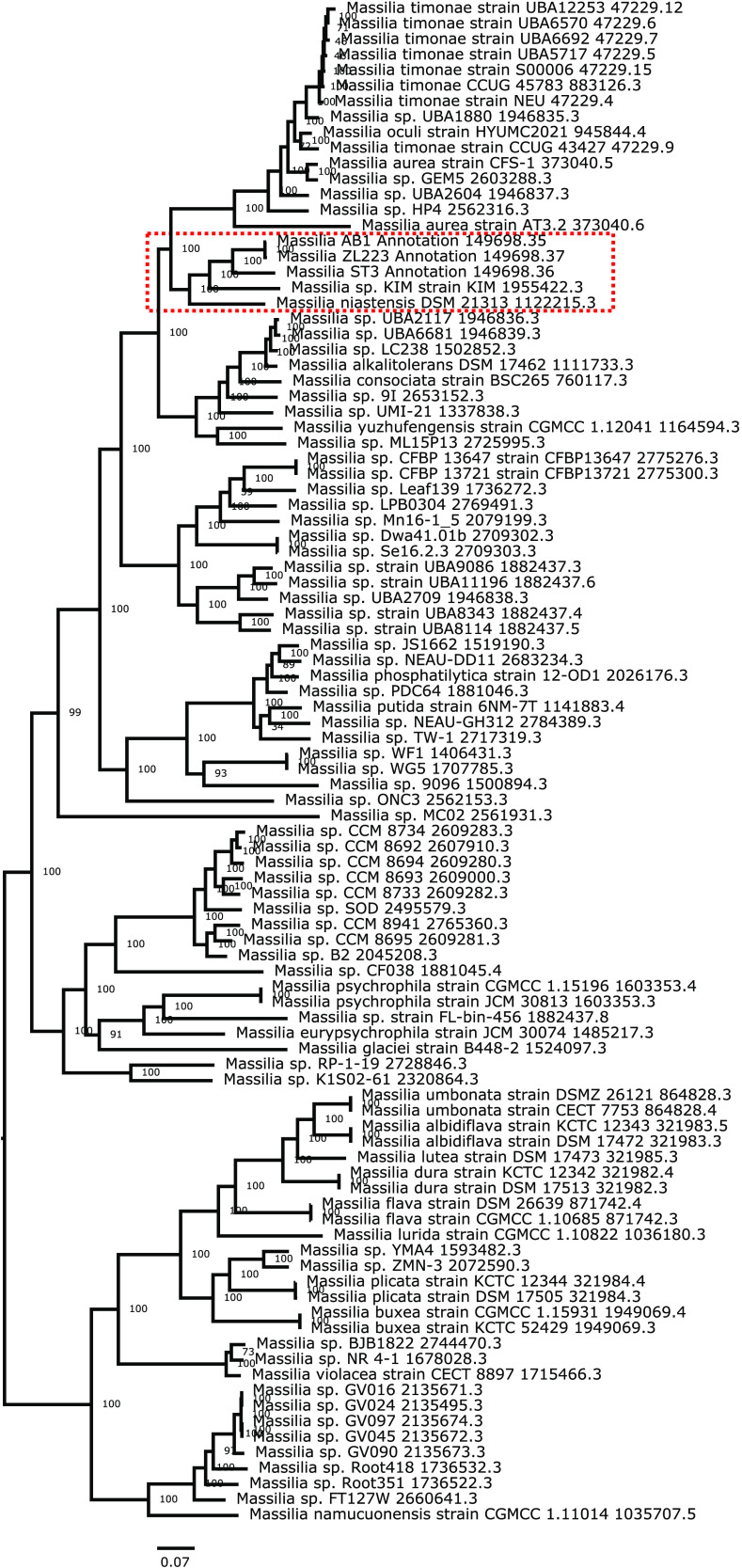
Assessment of *Massilia* sp. strain AB1, ST3, and ZL223 relatedness. Ninety-five complete, good-quality *Massilia* genomes from the PATRIC genome database and AB1, ST3, and ZL223 were analyzed using the Codon Tree pipeline in PATRIC. Forty single-copy genes, representing 10,078 amino acids and 30,234 nucleotides, were aligned, and support values were generated using 100 rounds of rapid bootstrapping in RAxML. No deletions or duplications were allowed. The red dashed box outlines the branch containing strains AB1, ST3, and ZL223.

To assess the relatedness of the three isolates, a pairwise comparison of the average nucleotide identity (ANI) (33) with 103 full *Massilia* genome sequences from NCBI was performed using the Kostas Lab ANI tool v1.0 with default parameters (34). AB1 and ZL223 shared 99% ANI, while strain ST3 shared 89% ANI with AB1 and ZL223. All three strains showed between 79 and 84% ANI with all other genomes queried, including 83% ANI with the type species Massilia timonae (GenBank accession number GCA_000315425.1). These results place strains AB1, ST3, and ZL223 within the *Massilia* genus but likely distinct species from those already reported. An unrooted phylogenetic tree generated using the Codon Trees pipeline (https://github.com/PATRIC3/codon_trees) in PATRIC (27, 35–39) showed AB1 and ZL223 as most closely related, followed by ST3 (Fig. 1, red box). Analysis by the Antibiotics and Secondary Metabolite Analysis Shell v6.0 beta, using the “relaxed” strictness setting (40), predicted 7, 4, and 6 biosynthetic gene clusters (BGCs) from strains AB1, ST3, and ZL223, respectively. Interestingly, only 3 of the 17 BGCs identified had any similarity to known clusters. Taken together, these strains add to the genomic data available for the *Massilia* genus and support further investigation into their biosynthetic potential.

### Data availability.

This whole-genome shotgun project was deposited at GenBank under BioProject accession number PRJNA719844. The GenBank assembly and Sequence Read Archive accession numbers are provided in Table 1.
